# Recovering Microalgal Bioresources: A Review of Cell Disruption Methods and Extraction Technologies

**DOI:** 10.3390/molecules27092786

**Published:** 2022-04-27

**Authors:** Md. Mijanur Rahman, Nushin Hosano, Hamid Hosano

**Affiliations:** 1 Graduate School of Science and Technology, Kumamoto University, Kumamoto 860-8555, Japan; 218d9362@st.kumamoto-u.ac.jp; 2 Department of Biomaterials and Bioelectrics, Institute of Industrial Nanomaterials, Kumamoto University, Kumamoto 860-8555, Japan; nushin@kumamoto-u.ac.jp

**Keywords:** microalgae bioresources, extraction, cell disruption, intracellular compounds, lipids, pulsed electric field

## Abstract

Microalgae have evolved into a promising sustainable source of a wide range of compounds, including protein, carbohydrates, biomass, vitamins, animal feed, and cosmetic products. The process of extraction of intracellular composites in the microalgae industry is largely determined by the microalgal species, cultivation methods, cell wall disruption techniques, and extraction strategies. Various techniques have been applied to disrupt the cell wall and recover the intracellular molecules from microalgae, including non-mechanical, mechanical, and combined methods. A comprehensive understanding of the cell disruption processes in each method is essential to improve the efficiency of current technologies and further development of new methods in this field. In this review, an overview of microalgal cell disruption techniques and an analysis of their performance and challenges are provided. A number of studies on cell disruption and microalgae extraction are examined in order to highlight the key challenges facing the field of microalgae and their future prospects. In addition, the amount of product recovery for each species of microalgae and the important parameters for each technique are discussed. Finally, pulsed electric field (PEF)-assisted treatments, which are becoming an attractive option due to their simplicity and effectiveness in extracting microalgae compounds, are discussed in detail.

## 1. Introduction

The world’s population is predicted to grow at a faster rate throughout this century, reaching 9.7 billion by 2050 [1]. To meet the demands of a rapidly expanding population, global food production needs to increase by approximately 70% over the next 30 years. In order to address the challenges associated with food security around the world, a multidisciplinary approach with regards to social, economic, and technological aspects of the global food supply is required. In view of the limited availability of raw materials, energy, nutrients, and water, a comprehensive strategy is required to overcome the shortage of food resources on a global basis [2]. These circumstances have led to the conclusion that new nutritional resources are necessary in order to meet the demands of the growing population. In this context, microalgae have long been considered a prominent source of a variety of metabolites and a significant store of nutrients such as protein, minerals, carbohydrates, vitamins, fats, and other organic compounds [3]. As well as providing a reliable source of nutrients, microalgae are also very well recognized as an untapped source of biofuel, which can be an important component to driving economic growth. Over the past few decades, microalgae for biofuel has been considered a promising alternative source of renewable energy that has the potential to replace fossil fuel in the near future [2]. 

Microalgae are a diverse group of unicellular photosynthetic organisms commonly found in marine systems and freshwater [4]. They contain a large number of organic compounds of potential value, such as amino acids, lipids, and polysaccharides. However, the gross compositions of some microalgal species may not always correspond to their nutritional value. For example, although the nutritional value of *Nannochloris atomus* and *Phaeodactylum tricornutum* is considered to be low, both of these species are rich sources of protein and carbohydrates, respectively [5]. In addition, the chemical composition of microalgae varies when they are cultivated under diverse environmental circumstances or harvested at different growing phases. Brown et al. [5] reported that culturing *prymnesiophytes Puuloua Zutheri* and *Zsochrysis* sp. in the logarithmic phase provided 30–50% more protein and 2–4 times less carbohydrates than in the stationary phase. 

Harvesting the microalgae is the next process after growth, which means detaching the algal biomass from the liquid medium [6,7]. An efficient harvesting technique for microalgal harvesting requires a rigorous step of removing water by various methods, including centrifugation, filtration, electrical-based processes and flotation [8,9]. After harvesting the microalgae, the most important step is to extract their compositions using appropriate techniques. In general, two methods are applied for the extraction of microalgal products: dry and wet routes. Dry route techniques include sun drying, drum drying, spray drying, and freeze drying before extraction. For the wet route, on the other hand, the harvested biomass is first disrupted in order to release the intracellular products. According to Patil et al. [10], dry route processing has a higher energy consumption than the wet route. Furthermore, based on currently available technologies, the wet route is more likely to produce high-value products than the dry route, particularly when it is used to produce biofuels [11]. In a wet route process, cell disruption procedures are usually performed prior to the extraction in order to facilitate the release of valuable biomolecules from the disturbed microalgae. 

An alternative to the disruption of the cells in order to gain access to the intracellular material would be to genetically modify microalgae that allow these materials to be released into the growing medium [11]. In view of the complex structure of microalgae, in which their genes are found in multiple locations (nucleus, chloroplast, and mitochondria), it is difficult to genetically engineer them, especially when compared to other microorganisms, such as bacteria. In spite of several genetic engineering techniques being developed for potential candidates, the enhancement of extracted products by genetic engineering is a challenging application that requires considerable investment in the future. 

Researchers and industry are becoming increasingly interested in microalgae that are capable of producing a sustainable assembly of a wide range of products with significantly faster growth rates, with the potential for process optimization and higher photosynthetic capacities, especially those that can be used in a wide range of applications such as food, cosmetics, animal feed, and other products. In this context, a thorough understanding of the characteristics of the microorganism cell wall can assist in determining the most appropriate method for disrupting the cellular integrity of the organism. The structure of algal cell walls is greatly influenced by factors such as the type of organism, the growth medium, the presence of certain chemical compounds in the culture, the composition of the growth medium, and external factors such as temperature. Therefore, the effectiveness of different destructive techniques may vary depending on the type of microalgae and their growth conditions [12]. In addition to determining the most appropriate technique, other considerations should also be taken into account, such as selecting a cost-effective growth medium and an energy-efficient harvesting method [12]. It is therefore necessary to conduct extensive research on microalgae cultivation, harvesting, and fractionation methods in order to receive more intracellular products.

The final stage of microalgal treatment in a wet route process is the extraction of its compounds. The optimization of a well-organized cell disruption process is an important step in maximizing the yield of microalgae biomass-based products, but it is also important to employ a realistic and energy-efficient process that allows for high quality, low operating costs, and greater extraction of microalgal products. Since the amount of hydrocarbons, protein, biofuels, and other materials harvested from microalgae highly depends on the process of cell disruption for the extraction, selecting the most suitable cell disruption method should be based on specific considerations ranging from the choice of microalgal strain to the quality of the extracted products, as well as the separation of contaminants. In addition, choosing an appropriate cell disruption technique is crucial to increasing the efficiency of the extraction from microalgae. For example, several cell disruption techniques are being used for the extraction of lipids from microalgae using the Folch method, the Bligh and Dyer method, the superior solvent extraction method, in situ lipid hydrolysis and the supercritical in situ transesterification (SC-IST/E) method, modification of the Folch/Bligh and Dyer method, and the mechanical method [13]. In addition, ionic liquid solutions are used as extractive solvents for the extraction of protein [14], carbohydrates and lipids [15], and biofuel [16]. Microalgal carbohydrates are extracted by means of supercritical fluid extraction (SFE) [17] and pressurized liquid extraction (PLE) [18,19]. However, SFE- and PLE-based carbohydrate extraction can be greatly affected by the biomass temperature [17,20]. Among various techniques, the combination of enzymatic and mechanical cell disruption methods provides an effective and efficient strategy for the extraction of a variety of the materials due to their independence from the type of microalgae [13]. Therefore, a range of cell disruption methods have been proposed by researchers in their pioneering research for the extraction of various compounds from microalgae.

The majority of cell disruption methods are primarily being developed to enhance the extraction yield of intracellular compounds. To date, however, commercial extraction from microalgae has not been developed due to the lack of a comprehensive understanding of the potential advantages and limitations of cell disruption methods. It is therefore necessary to further investigate the currently available methods of cell disruption for microalgae in order to fully utilize them as a source of various value-added compounds. This review article provides an overview of the conventional cell disruption methods and their fundamentals, physics, and potential applications. The search was conducted using keywords related to microalgae and cell disruption methods based on data found in the Kumamoto University library database, patent research, and Google Scholar. The study involved the review of over 300 peer-reviewed publications (articles, books, or sections of books) in the field of microalgae extraction methods. As a result, the technical efficiency of the major extraction processes, their industrial implementations, and their future prospects within the market were evaluated. The article examines and compares all methods regarding their efficiency in disrupting cells and their prospects for facilitating extraction from microalgae. Moreover, a special focus has been placed on the recovery of various products by means of the PEF-treated technique after initial cell disruption. A detailed review of this technique is separately presented at the end of the review for further implementation.

## 2. Cell Disruption Methods

**Figure 1:**
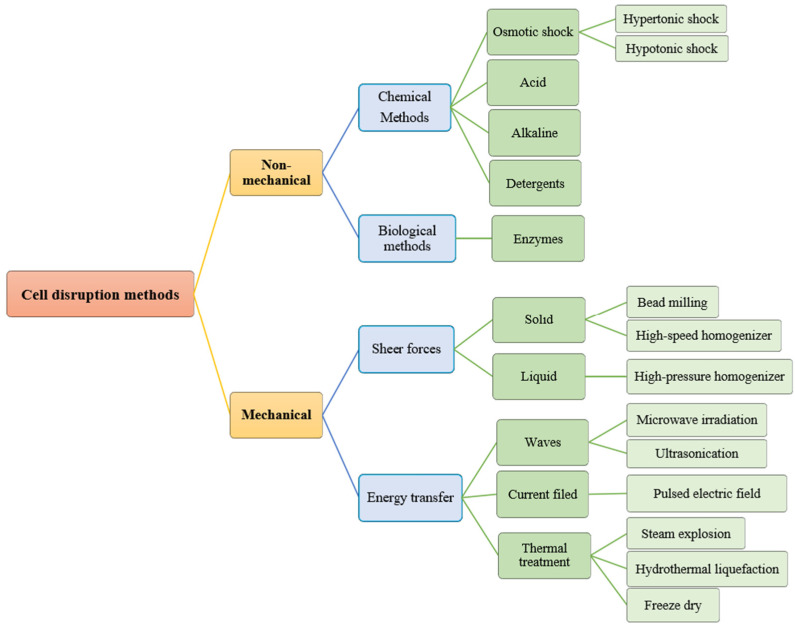
Different cell disruption techniques for microalgae.

In general, cell disruption methods can be broadly classified as non-mechanical and mechanical approaches [12]. Each group can be further divided into the methods shown in Figure 1.

### 2.1. Non-Mechanical Methods 

In this section, we will describe common non-mechanical methods that can be used to disrupt the cell wall of microalgae in order to allow the release of their intracellular biological molecules. Non-mechanical methods include osmotic shock [21,22], chemical methods [23,24], enzyme treatment [25], and detergents [26]. 

#### 2.1.1. Osmotic Shock 

Osmotic shock refers to the process of applying a high concentration of a solute, such as dextran, salts, or polyethylene glycol, around a cell in order to reduce its osmotic pressure. This hostile environment can cause damage to the cell wall of the microalgae, thus releasing their intracellular compounds. Besides, hypotonic osmotic shock is able to damage the cell membranes of almost all species of microalgae but is unable to rupture the cell walls [22]. 

Osmotic shock is used as non-mechanical cell disruption technique owing to the extraction of lipid [27], proteins [28], and carbohydrates [29] from microalgae. In their study, Rakseh et al. [30] compared the osmotic shock technique with different methods of cell disruption, such as autoclaving, pasteurization, and microwaving, for the extraction of lipids from *Chlorella sorokoniana* MIC-G5, *Chlorococcum* sp. MCC30, *Botryococcus* sp. MCC32, and *Botryococcus* sp. MCC31. They claimed that the highest lipid extraction could be achieved by the osmotic shock technique for *Botryococcus* sp. MCC32 (15% NaCl) and for *Chlorella sorokoniana* MIC-G5 (5% NaCl). Gonzalez-González et al. [31] also used the osmotic shock technique to extract lipids from two lipid-rich microalgal strains, *Chaetoceos muelleri* and *Dunaliella salina*, achieving extraction efficiency values of 72% and 21%, respectively. Therefore, they recommended osmotic shock as a suitable method of lipid extraction from *Chaetoceos muelleri*, but not *Dunaliella salina.* Osmotic shock has also been applied for the extraction of lipids together with other materials, such as biodiesel, biogas [31], and palmitate [30]. According to a study that used the osmotic shock method for simultaneous lipid and methane extraction, this method was able to release 72% of microalgal lipids and produce 35% of methane from *Chaetoceros muelleri* algae [32]. 

In addition, osmotic shock has been used to disrupt microalgal cells for the purpose of protein extraction. The use of osmotic shock in combination with ultrasound pretreatment and enzymes for the extraction of protein from *Chlorella vulgaris* has been optimized by adjusting key parameters such as enzyme concentration, treatment time, and extraction duration [28]. Furthermore, the liquid biphasic flotation-based osmotic shock technique has been employed for the recovery of entire proteins from *Chlorella vulgaris* FSP-E. Different parameters affecting the protein recovery, including microalgae biomass concentration, air flow rates, types of salt, types of alcohol, concentration of alcohol, concentrations of salt, and osmotic shock period, have also been studied [33]. After optimizing the parameters, a total of 92.98% protein was recovered along with a separation efficiency of 64.91%. Hence, this technique has been recommended for large-scale implementation in terms of protein recovery and sustainability. 

Microalgal carbohydrates are another important intracellular compound that can be extracted with the assistance of osmotic shock treatment. Bremauntz et al. [29] demonstrated the extraction of carbohydrates from two types of microalgae, *Chlorella vulgaris* and *Scenedesmus* sp., using osmotic shock. After 24 h of osmotic shock, the latter strain showed a significant increase in the production of carbohydrates with 0.1 M NaCl. According to the results, the amount of carbohydrate extracted through the osmotic shock treatment process was proof of the effectiveness of the osmotic shock treatment.

In conclusion, the ease of scaling up, lower input energy consumption, and modest capital cost made the osmotic shock technique attractive to researchers and industrialists [34]. However, sometimes this method is time-consuming and ineffective at extracting microalgal compounds when microalgae cells are rigid [35]. 

#### 2.1.2. Chemical Method

Cell disruption can occur by chemical methods as a result of the interaction between microalgae and a variety of chemicals, such as organic solvents, surfactants, hypochlorite, and chelating agents. The optimum contact time and the concentration of reactant have been investigated in the cell disruption of *C. vulgaris* using only H_2_O_2_ and a combination of H_2_O_2_ and FeSO_4_ [36]. When H_2_O_2_ and the combination H_2_O_2_ + FeSO_4_ were used as the extraction medium, lipid extraction increased from 6.9 to 9.2% weight per weight (*w*/*w*) and from 6.9 to 17.4% (*w*/*w*), respectively. To improve the lipid extraction efficiency from microalgae *Scenedesmus* sp. employing the enzyme-aided chemical extraction technique, various parameters (cellulase, pectinase, xylanase, enzyme concentration, pH, temperature, and incubation time) were examined and optimized [37]. A total of 96.4% lipid was extracted, applying cellulase (20 mg g^−1^), pectinase (10 mg g^−1^), and xylanase (14 mg g^−1^) with a pH value of 4.4 and a temperature of 45 °C for a duration of 190 min.

Cell wall disruption and protein extraction have also been studied for *Chlorella sorokoniana* and *Chlorella vulgaris* based on solvent types, ultrasonication, and alkalis techniques. The combination of ultrasonication and alkalis treatment has revealed the highest cell disruption efficiency, and the total extracted proteins from *Chlorella sorokoniana* and *Chlorella vulgaris* have been estimated to be 19.88 ± 0.38% and 27.68 ± 0.53%, respectively. Releasing protein from microalgae, however, needs a method capable of effectively disrupting the rigid cell walls. This challenge can be overcome by applying chemical techniques in combination with mechanical techniques [24]. The effects of different types of solvents, including alcohols and water, have been investigated for the extraction of protein. Water is recommended as an appropriate extractive solvent in industrial-scale application due to the benefits of being low-cost, presenting minimal safety risks, and being globally available. A combination of chemical and mechanical techniques is applied for the cell disruption of microalgae strain *Chlorella vulgaris* to extract protein and carbohydrates [38]. 

The chemical treatment, particularly base-catalyzed, was demonstrated to be an effective technique for cell disruption when up to 56 weight (wt)% of protein and 14 wt% of carbohydrates of total biomass were extracted. A new technique called the cationic surfactant-based harvesting and cell disruption approach has also been investigated to determine its efficiency on harvesting microalgal biomass and cell disruption for the production of biodiesel [26]. Using this technique, a total of 91% harvesting efficiency was obtained after only 5 min, whereas for 97% efficiency the duration was prolonged to 90 min. Notably, all of these results were obtained without using any energy-intensive apparatus. 

As a result, chemical-assisted techniques for microalgal cell disruption are widely used because of their simplicity, convenience for continuous operation, and suitability to diverse production scales [39]. Nonetheless, longer extraction times and higher energy consumption are considered to be the principal challenges of chemical-assisted cell disruption [40].

#### 2.1.3. Enzymatic Method

Enzymatic cell disruption is a highly selective technique for the recovery of microalgae intracellular compounds that is eco-friendly and modest in lipid product extraction compared to solvent-based techniques [41]. Zheng et al. [41] presented an effective enzymatic-based cell wall disruption technique by combining with thermoresponsive polymers, such as pNIPAM and p(NIOAM0co-AA), to recover the lipid contents and reduce sugar. They found that the polymers were capable of disrupting microalgal cell walls but hydrolyzed cell walls. Lysozume, cellulase, glucanase, and protease are being used as commercial enzymes and generally employed in the immobilized form to enhance disruptive stability and durability in catalytic action [42].

A combination of enzymes, including galatomannanase, β-glucosidase, and 1,4-β-cellobiosidase, has been used for the cell wall disruption of the marine microalgae *Nannochloropsis* sp., and factors affecting the lipid extraction have also been studied [43]. Only 70% of total lipids has been recovered using the parameters of a treatment time of 90 min, an enzyme dosage of 1.3 mg g^−1^, a pH value of 5, and a temperature of 36 °C. Liang et al. [44] demonstrated the combination of enzyme-assisted aqueous extraction processing (EAEP) with ultrasonication for cell disruption and lipid extraction using three different types of microalgae (*C. vulgaris*, *Scenedesmus dimorphus*, and *Nannochloropsis* sp.). Lipid recovery is greatly affected by enzyme type, incubation time, pH, enzyme dosage, and type of algae. Having completed a comparison among various enzymes, the highest lipid recovery of 49.82% was achieved by snailase and trypsin from *C. vulgaris*, whereas this recovery was 46.81% and 11.73% from *Scenedesmus dimorphus* and *Nannochloropsis* sp., respectively. Zhang et al. [37] extracted 96.4% of lipids from microalgae *Scenedesmus* sp. by applying a mixture of enzymes (xylanase, pectinase, and cellulase) using various parameters, such as incubation time, temperature, pH, and enzyme concentration. Though the lipid extraction was improved from *Scenedesmus* sp. in the works by Zhang et al. [37], the enzymes were employed just as pretreatment followed by an organic solvent extraction, whereas Liang et al. [44] used a more appropriate technique based on EAEP [44]. A combination of enzymatic and bead-milling techniques have been developed for the extraction of proteins, lipids, and carbohydrates from microalgae *C. vulgaris* using enzymes such as lipase, cellulase, protease, and phospholipase [45]. They recovered 88% lipids, 74% carbohydrates, and 68% proteins without losing any products, reducing residence time and avoiding any corrosive solutions. Soto-Sierra et al. [46] presented an aqueous two-step enzymatic treatment for the extraction of internally stored lipids from *Chlamydomonas reinhardtii* microalgae using autolysin and trypsin enzymes. They found 50.1 ± 4.2% of proteins from the primary treatment with autolysin and 73 ± 7% of lipids from the secondary enzyme treatment with trypsin for chloroplast. 

The enzymatic disruptive technique has produced hopeful results for extraction from yeast in laboratory, confirming the peptidoglycan structure of the cell wall [47]. Low operational temperature is required in this method and has high selectivity of lipid class and no related corrosion matters, making it a desired method for microalgal cell wall disruption [48]. However, the longer processing time, possibilities of product inhibition, and lower production capabilities for cell disruption of microalgae are the main drawbacks to the enzymatic technique compared to the mechanical and chemical techniques [49]. Moreover, the high price of enzymes limits their application in microalgal extractions. 

#### 2.1.4. Detergent Method 

The cell wall of microalgae can be disrupted using the detergent method (acid and/or alkali). Detergents are used for weakening the cell membrane to release metabolites and enhanced the diffusion of solvent that can extract the compounds out of the cell. The detergent method has been applied for the cell disruption of various microalgae [50,51,52]. The sulfuric acid treatment is applied as a non-mechanical technique for the disruption of *Chlorococcum* sp. using different temperatures, acid concentrations, and treatment periods [51]. A higher degree of microalgal cell disruption was observed at higher temperatures (160 °C) and longer treatment durations compared to the same treatments at lower temperatures (120 °C). However, regardless of its higher efficiency in microalgal cell disruption, there is a possibility of destroying the intracellular compounds, such as pigments and proteins, due to the acidic action. Acid treatments offer a cost-effective and lower-energy-consumption technique for microalgal cell disruption but are greatly affected by the temperatures required for the extraction [50]. The alkaline treatment of microalgal biomass was applied for the production of bioethanol from microalgae species *Chlorococcum infusionum* [53]. The effects of three parameters—treatment duration, concentration of NaOH, and temperature—on bioethanol production were investigated in that study. Irrespective of having good results after treatments, the authors recommended further studies of the alkaline-based microalgal treatments. This technique can be scaled up easily, but needs a special sort of reaction system. Moreover, further processing of the product stream is essential for the removal and neutralization of the chemicals from the extracted compounds [52]. 

### 2.2. Mechanical Methods

Mechanical methods are alternative techniques to disrupting cells through the application of forces in the form of solid and liquid shearing (e.g., bead milling, high-speed homogenizer, and high-pressure homogenizer), in the form of energy transfer (e.g., ultrasonication, microwave, and laser), as heat (e.g., thermolysis and autoclaving), or as a current (e.g., pulsed electric field) [54]. In order to select the most appropriate method for microalgae cell disruption, it is important to consider the following factors: the nature of biomolecules, the scalability, the input energy, the cell wall composition and the concentration of biomass.

#### 2.2.1. Bead Milling/Bead Beating

**Figure 2:**
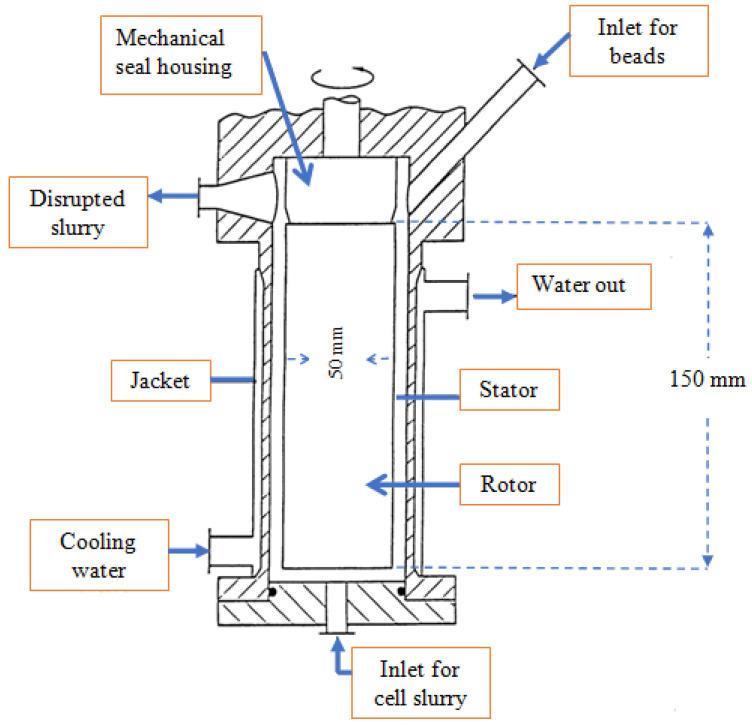
A schematic diagram of the bead mill disruption chamber [56]. Adapted with permission from Springer Nature (1998 Springer-Verlag).

In the bead milling, particles are ground and dispersed into micro or nano size by grinding and dispersion machines. In this technique, the cells are damaged by applying the force through the collisions between the cells and the beads. With the help of a rotating shaft in the grinding chamber, the collision is propped up. The effectiveness of microalgal cell lysis by this technique primarily depends on the load of the beads and their diameter [55]. To apply the multiple collisions in the chamber, glass or ceramic beads can be selected [49]. Zirconium and glass have been used in this process as high- and low-viscosity media, respectively. A schematic diagram of the equipment along with a perpendicular bead-milling chamber for bioplastic poly recovery from *Alcaligenes latus* is presented in Figure 2.

In commercial-scale applications, the use of solid or liquid shear forces is generally more common owing to their higher efficiency and easier scale-up. An optimized mechanical technique was proposed for cell wall disruption of *Chlorella* at industrial scale [56]. In this study, the diameter of milling beads was considered in a range of 0.3 mm to 1.7 mm, zirconium silicate beads with a density of 2 to 3.2 Kg L^−1^, a milling chamber volume ≥ 500 L, a flow rate ≥ 1 m^3^/h, a chamber filling rate of 80–90%, and a batch from 1 to 200 m^3^. This arrangement was recommended as the optimized multiple pass mode operation, along with higher productivity and lower energy consumptions for cell breaking of microalgae. Bead milling has been proposed as an efficient and placid disintegration method [57]. Using smaller-size beads, the energy consumption was reduced by 1.2 kWh kg^−1^ DW; however, the production yield remained unchanged. Thus, regardless of having numerous advantages, including high product quality and comparatively low energy consumption, the bead-milling technique still needs to increase its effectiveness [58]. The amount of microalgal compounds extracted after employing bead beating as a cell disruption technique is tabulated in Table 1, considering various operating parameters of the applied method along with the microalgal species. It will help the reader to apply this technique considering various process parameters as well as targeting the extracting products from the microalgae.

#### 2.2.2. High-Speed Homogenization

High-speed homogenization (HSH) is a very effective and simple method for breaking microalgae cells with the assist of dynamic cavitation. The slurry of biomass is stimulated in a precise apparatus consisting of a stator–rotor assembly, where the gap is considered to be as small as 100–3000 µm [70]. Generally, the HSH is made of stainless steel, where the stators and rotors are designed in a multiplicity way. To extract lipids and antioxidants from *Nannochloropsis* sp., *Phaeodactylum tricornutum*, and *Pavlova lutheri*, the required operational time was mentioned as 30 s and 60 s at a speed of 10,000 rpm and 14,000 rpm, respectively [71,72]. HSH was applied for cell disruption and lipid extraction simultaneously for algae *Aurantiochytrium* sp. wet biomass with a higher shear mixer. Hence, 80% of lipids was extracted after 10 min when stirred at a speed of 15,000 rpm [73]. A comparative study was conducted among five oil extraction techniques along with HSH for five microalgae strains (*Guinardia* sp., *Navicula* sp., *Closterium* sp., *Nannochloropsis* sp., and *Amphiprora* sp.) at lab scale [74]. The highest average extraction efficiency belonged to the HSH technique when this system was operated by the combination of polar/non-polar solvents. The shear forces were applied at the solid–liquid interface with high-speed stirring from 10,000 rpm to 20,000 rpm [54]. However, this method has some specific disadvantages (e.g., high energy consumption and denaturation of protein), and it is fit mainly for application at the industrial scale when a short processing time is required.

#### 2.2.3. High-Pressure Homogenization

High-pressure homogenization (HPH) is a mechanical technique that is used to diminish particle size or to lyse cells by applying the highly pressurized liquid (20 to 120 MPa) through a thin nozzle. The number of homogenization passes and the operating pressure are two controlling parameters that should be optimized to enhance the efficiency of this technique [42]. The vulnerability of microalgae strains *Nannochloropsis* sp., *Tetraselmis suecica*, and *Chlorella* sp. to cell disruption by HPH were compared at industrial scale, taking into consideration parameters including cell density, metabolite, turbidity, and particle sizing [75]. Cell counting was used to investigate the cell rupture for all types of microalgae. It was shown that *Tetraselmis suecica* could easily be ruptured, whereas *Nannochloropsis* sp. had the highest resistance to rupture through HPH. Lipid extraction from *Chlorella saccharophila* using HPH along with three-phase partitioning was presented by Spiden et al. [76], wherein the cells were disrupted by probe sonication. The parameters of HPH for cell disruption was optimized (temperature: 25 to 35 °C, time: 60 min and slurry: t-butanol = 1:0.75) and finally 89.91 ± 3.69% (*w*/*w*) lipids, 1.26% (*w*/*w*) carotenoids of dry biomass, and 12% (*w*/*w*) protein of dry biomass were obtained [77]. A comparative study was carried out among six different techniques, including HPH for the cell disruption of microalgae *Desmodesmus* sp. F51 for the optimization of carotenoids [78]. Considering cell density (2.0–90 g L^−1^), pressure (10–40 kpsi), and homogenization cycles (1–4), the HPH model provided well-fitted disruption performance. 

**Figure 3:**
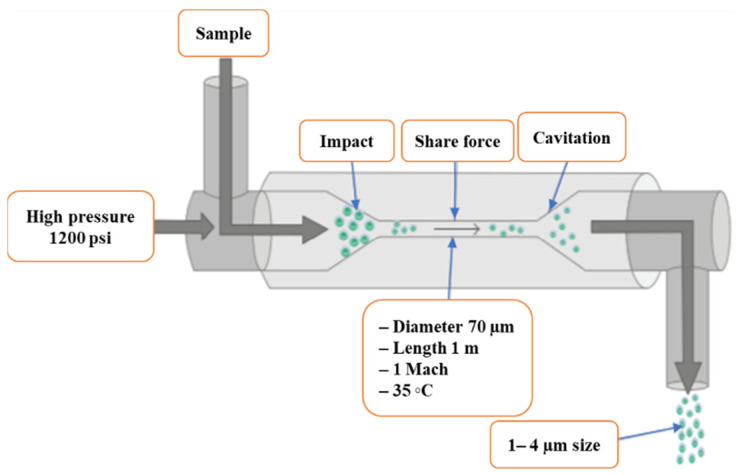
A schematic diagram of a high-pressure homogenizer used for the cell disruption of microalgae [79]. Adapted with permission from Hindawi (Creative Commons Attribution License).

A schematic diagram of HPH for the cell disruption of marine microalgae *Scenedesmus* sp. is shown in Figure 3. The ultra-HPH technique was evaluated for cell disruption of *Nannochloropsis* sp. suspension with other three methods [79]. The number of homogenization passes was inversely proportional to the ultra-high pressure (250 MPa) for the specific degree of cell disruption.

However, heat produced by ultra-high pressure after several homogenization cycles resulted in enormous aggregate configuration of released intracellular substance [80]. Mechanical cell disruption was investigated for microalgal strains *Tetraselmis suecica* and *Chlorococcum* sp. using ultrasonication and HPH, and the techniques’ performance was compared [81]. This study concluded that the rate of disruption was directly proportional to the operating pressure, whereas it was inversely proportional to initial concentration of the cell. The effects of cell disruption techniques on the diffusion of *C. vulgaris* was examined for intracellular extraction considering four different techniques, including HPH [66]. The highest number of extracted proteins and pigments was obtained from HPH rather than other techniques. Cell disruption occurred partially while a notable number of cells remained intact. Hence, this method for the cell disruption of microalgae is still in its early stages and needs further in-depth research.

#### 2.2.4. Microwave Irradiation

Microwave irradiation (MI) is an environmentally friendly technique generally employed for organic synthesis [82]. MI is a straightforward and scalable practice for microalgal cell disruption. The cell walls are disrupted electromagnetically, which is induced by MI. It acts together with the dielectric and the polar (water) molecules, and as a result heat is produced. The parameters were optimized for heating, drying, and cell disruption of microalgae using MI at a time of 90 s, power of 800 W, and frequency of 2450 MHz [83]. MI technology was applied for the disruption of *Chlorella* sp. cells at a high temperature of 100 °C, frequency of 2450 Hz, and time duration of 5 min [63]. The volumes of cell suspension used in these studies were 250 mL and 100 mL, respectively, and 18% and 11.2%, respectively, of lipid concentration of *Chlorella* sp. was obtained. A continuous microwave technique was designed and optimized for the extraction of green algal oil from *Scenedesmus obliquus* [84]. The cell suspension was prepared with the same ratio of algae and water and was heated up to 95 °C for 30 min. As a result, a total of 76–77% oil was extracted by a microwave system at 95 °C applied for 20–30 min. Different cell disruption methods including MI have been investigated for the lipid extraction of *C. vulgaris* biomass [85]. A suspension was prepared by blending 4 gm of dry cell biomass and 800 mL of distilled water and the mixture was placed in a household microwave oven (2.45 GHz and 800 W) for only 15 min, and four extracting techniques were applied. The highest percentage of lipids (82.87%) was extracted with the microwave irradiation method. Hence, MI has been categorized as the most effective, simple, and easy method for microalgal lipid extraction. 

A combination of various mechanical and chemical methods (microwave irradiation, ultrasonication, grinding with liquid N_2_, osmotic shock, cooling, and freeze-drying) for cell disruption of *Scenedesmus* sp. have been compared for the extraction of neutral lipids and free acids [86]. Introducing liquid CO_2_ appeared as a novel and greener extraction technique due to using a low pressure of 150 bar, 25 °C temperature, and lower capital cost. Total lipid (13.2 wt%) was acquired from the Soxhlet extraction, whereas 9.6 wt% lipid was obtained by applying microwave radiation alone. Moreover, microwave irradiation was compared with four different methods, including bead beating, autoclaving, sonication, and 10% NaCl solution, to select the most effective cell disruptive technique for three different strains of microalgae, *Botryococcus* sp., *C. vulgaris*, and *Scenedesmus* sp. [61]. Lipid extraction efficiency was examined according to different extraction techniques and the type of microalgae. The *Botryococcus* sp. strain depicted the highest lipid extraction when the microwave oven technique was applied. The performance of microalgal cell wall disruption depended on the microwave treatment temperature, treatment duration, cell wall thickness, and pore diameter [84]. Microalgal cell fractal dimension increased by 0.3 µm by enhancing the temperature of the microwave treatment by 40 °C. Moreover, due to the increasing the duration of the treatment from 0 to 20 min, the pore diameter and cell wall thickness increased by 0.175 µm and 0.48 µm, respectively. 

The amount of lipid extraction after employing microwave irradiation as a cell disruption technique is tabulated in Table 2, considering various operating parameters of the applied method along with the microalgal species.

#### 2.2.5. Ultrasonication

Ultrasonication comprises prompt compression and decompression successions of sonic waves. This continuous cycle produces cavitation inside the cell, which contain liquid vapor called microbubbles. Due to the acoustic waves of the liquid molecule movement, the microbubbles are formed. According to the intensity of ultrasound, microbubbles are compressed and then collapse. Hence, they produce heat, high pressure, free radicles, shockwaves, and, eventually, destroy the cell walls [94]. A wide range of ultrasonic power (0–50 W), ultrasonic time intervals (0–5 s), and exposure time (0–10 min) was applied to improvement lipid and biomass content [95]. When the optimum parameters were selected as ultrasonic power, 20 W; ultrasonic time interval, 2 s; and a 4 min ultrasound, the lipid production increased from 0.76 to 1.31 g L^−1^. The ultrasound treatment enhanced the release of intracellular components, but did not affect the cell wall integrity [96]. Microalgae have been harvested by chitosan to assist with biodiesel production and lipid extraction using ultrasound treatment [97]. The highest harvesting efficiency of about 97%, lipid extraction yield, and biodiesel production have been achieved with chitosan-harvested biomass. The two-stage treatment of *C. sorokoniana* SDEC-18, fast cell growth, and improve lipid productivity was studied by seawater and ultrasound [98]. In the first stage, ultrasound-aided lipid extraction reached up to 64% and a lipid productivity of 28.78 mg L^−1^ DCW was obtained in the second stage. 

Ultrasound pre-treatment facilitated the extraction of protein from *Ascophyllum nodosum* with acid or alkaline treatment by 540% and 27% with and without pretreatment, respectively. Hence, the processing time was reduced by 50 min [99]. Ultrasonication in *C. vulgaris* extensively improved the crude protein digestibility over untreated spray-dried and electroporated *C. vulgaris* by 56.7 ± 13.7%, 46.9 ± 12.7%, and 44.3 ± 7.5%, respectively [100]. In addition, no bad impact was found when feeding green microalgae to animals. To further improve the protein extraction, an alternating ultrasonic frequency of 15 and 20 kHz has been recommended for *Prophyra yezoensis* as a viable method [95]. As a result, yields from microalgae were increased by 50% and 18%, and the extraction time was reduced, as well. Protein recovery along with umami free amino acids from *Chlorella vulgaris* was proposed by introducing ultrasound-aided single solvent extraction, sequential solvent extraction, and enzymatic extraction [65]. The ultrasound-aided enzymatic extraction method promoted protein recovery from 58 to 80% with a low percentage of umami free amino acids. 

A combination of ultrasound and ozone parameters was applied to recover proteins, lipids, and carbohydrates from microalgal biomass *Desmodesmus* sp. [101]. High cell concentration increased the cell disruption with low ultrasonic intensity. Almost 92% solvent waste and 80% of extraction time was reduced for both the treatment parameters. Three different microalgae species (*Spirulina platensis*, *Nannochloropsis gaditana*, and *Scenedesmus almeriensis*) were considered to investigate the effectiveness of the extraction of protein, lipids, and carbohydrates by means of ultrasound treatment [102]. With the same cell disruption technique parameters (ultrasound DCW 0.4 g L^−1^, time: 5 min, 24 kHz, and 400 W), this article concluded that *Spirulina platensis* was mainly composed of protein (~27%), whereas *Nannochloropsis gaditana* produced 33% carbohydrates and 41% lipid and *Scenedesmus almeriensis* produced 44% carbohydrates and 32% proteins.

A response surface methodology was employed to optimize the extraction of proteins, pigments, carbohydrates, and antioxidants using ultrasound treatment [103]. The optimum conditions were proposed as 30 min, 50 °C, and a pH of 8.5 for the extraction of nutrients, bioactive compounds, and antioxidant capacity. A continuous physical method of low-frequency non-focused ultrasound was applied to investigate the cell disruptive effectiveness of microalgae *Chlorella* sp. [104]. The most efficient cell disruptive conditions were determined considering the initial concentration, processing volume, and initial pH value of the microalgal cells. The physical ultrasound-assisted method was proposed for extracting carbohydrates from microalgae considering different operating parameters—biomass concentration, microalgae strain, extraction time, amplitude, and configuration of the ultrasonication constraints [105]. The highest extractions were obtained using 90 µm of amplitude and an ultrasonic pulse of t_on_/t_off_ = 0.2 W. A different method, an ultrasound-aided liquid biphasic flotation (LBF), was applied for the cell disruption of microalgae *H. pluvialis* [106]. Optimized values of different operating parameters of the ultrasound-aided LBF system, such as the ultrasound horn position, mode and amplitude of ultrasonication, flow rate of air, duration of air flow rate, and mass of *H. pluvialis*, were suggested. The effect of ultrasonic power, frequency, pressure, and temperature were inspected on the bubble motion of ethanol cultivation based on the Rayleigh–Plesset equation [107]. Consequently, the algae oil extraction rate obtained was 93.76 ± 0.48% for the application of ultrasonic power 150 W; the reaction required 30 min exposure at a temperature of 50 °C. Ultrasound-aided treatment increased the recovery of bioactive compounds from the microalgae *Nannochloropsis* sp. with a binary mixture of solvents [108]. Kadam et al. [109] proved that the ultrasound-assisted method could extract double the yield of typical water extraction. Ultrasound treatment is effective for disintegrating microalgal cells, and it enhances the concentrations of carbohydrates, protein, and lipids while at the same time decreasing the biomass concentration. The optimum energy intensity of 0.4 kWh L^−1^ was determined in the wastewater treatment using ultrasound. An ultrasound-assisted extraction was offered for zeaxanthin from *D. tertiolecta* considering ultrasonication time, temperature, and solid–liquid ratio [110]. Eventually the three parameters were finalized as an ultrasonication time of 33.6 min, temperature of 59.2 °C, and algal biomass–solvent ration 1:62 (g mL^−1^). 

The amount of microalgal intracellular extraction after employing ultrasonication as a cell disruption technique is tabulated in Table 3, considering the various operating parameters of the applied method along with the microalgal species. 

#### 2.2.6. Thermal Treatments

Thermal treatments are physical techniques where heat is employed to uphold cell disruption in the forms of steam explosion [117,118], thermolysis [119], and autoclaving [63,92]. Four cell disruption techniques (steam explosion, ultrasonification, autoclaving, and microwave irradiation) have been investigated to find the most effective method for the extraction of microalgal compounds from three different strains of microalgae (*Nannochloropsis gaditana*, *Phaeodactylum tricornutum*, and *Chlorella sorokoniana*) [92]. Among these tested methods, the highest amount of lipids was obtained by applying the steam explosion method on all microalgae types. An experiment was carried out by applying steam explosion as a cell disruption technique for the microalgal species *Nannochloropsis gaditana*, *chlorella sorokoniana*, and *Dunaliella tertiolecta* for a duration of 5 min at 150 °C [117]. The total operating cost depended on the cost of fuel, and it varied from 0.005 USD/kg^−1^ to 0.014 USD/kg^−1^ per microalgae dry sample. Moreover, the membrane filtration cost was anticipated as 0.12 USD/kg^−1^ per microalgae dry sample. Two techniques (thermal lysis and enzymatic lysis) were applied simultaneously for the cell disruption of *Nannochloropsis oceanica* at a steam pressure of 0.4 MPa, flow rate of 50 mL s^−1^, temperature of 121 °C, and duration of 20 min [120]. This study proved that lipids and proteins could be extracted by a single extraction process with a lower extraction cost when the total extracting lipids with moisture content was 96.0%. 

Among the thermal cell disintegration techniques, the autoclaving method has been widely used due to the lower maintenance cost [34]. Florentino de Souza et al. (2014) suggested that the autoclaving technique was more effective than ultrasonication for microalgal cell disruption on lipid extraction [121]. The autoclaving technique has been tested for the cell disruption of *Botryococcus* and *Chlorella* sp., where this method was found to be ineffective due to the lipid extraction of only 1% and 2.5%, respectively. However, steam explosion was found to be an effective technique for lipid extraction from *Nannochloropsis* [54]. In spite of having a low cost and simple operation, this technique is considered to have lower efficiency and higher energy consumption, and to produce of large amounts of unexpected cell fragments. The amount of lipid extraction due to employing autoclaving as a cell disruption technique is tabulated in Table 4, considering the various operating parameters of the applied method along with the microalgal species. 

#### 2.2.7. Pulsed Electric Field

The pulsed electric field (PEF) is a physical technology that involves the application of an electric field for a brief period of time, from nanoseconds to microseconds [123]. The electric pulse causes the cell membrane to break and create temporary or permanent holes or pores. Using this system, it is possible to access the intracellular components of microalgae. In recent years, PEF treatments have gained popularity as a cell-disrupting technique for microalgae. Further studies have, however, revealed that it could also be used in the extraction of proteins and lipids for commercial purposes [124]. Goettel et al. [124] were the first to propose using PEF as a technique for the extraction of intracellular components from microalgae. Their work proved that cell disintegration does not depend on the variation of the electric field but on the variation of specific energy inputs. Furthermore, the energy required for cell disintegration of 100 g dry weight per kg suspension (g DW kg^−1^ sus) was only 1 MJ kg^−1^ DW in the PEF treatment. Since then, PEF technology has been demonstrated to extract several microalgal compounds, such as lipids and carbohydrates [124]. Zbinden et al. [125] offered a promising downstream method for PEF treatment of microalgae and claimed that the conductivity of the microalgae suspension was increased due to this treatment. In addition, the energy requirement for the wet extraction of lipids from PFE-treated biomass was only 1.5 MJ kg^−1^ DW, whereas from drying biomass this power increased to 7 MJ kg^−1^ DW. Chloroform and methanol are advanced solvents for the extraction of lipids from microalgae due to their ability to overcome the resistance provided by the cell walls and membranes. However, these solvents are too expensive and toxic compared to PEF [126]. Furthermore, the extraction of non-lipid materials can be minimized using PEF treatment, and the fatty acid methyl ester (FAME) profile remains unchanged. Lai et al. [127] studied the impact of electric field strength, specific energy input, and cell disruption rate for PEF-aided lipid extraction of *Chlorella*. The authors reported that the lipid extraction yield could be controlled by varying the PEF parameters, and lipid extraction yields could be increased by up to 166.67% with PEF treatment.

PEF-assisted treatment was applied in *C. vulgaris* to demonstrate the influence of various parameters on protein extraction [128]. About half of the proteins could be extracted through PEF-assisted treatment, and the extraction effectiveness was greatly influenced by the biomass concentration, incubation time, temperature, and pH of the microalgae suspension. It was found that during the PEF process, microalgae were inclined to precipitate on the surface of the treatment electrodes [129]. The precipitation impacted the treatment process by distorting the electric field and the flow of microalgae through the system. Various processing parameters of PEF treatment have been examined on the extraction of precious compounds from microalgae species *Chlorella vulgaris* [130]. The effect of variation of the field strength (27–35 kV cm^−1^) and input energy (50–150 kJ kg^−1^) have been investigated in a PEF experiment at laboratory scale that determined the enhancement of the time–conductivity profile [131]. Moreover, the phenolics of the supernatant and the amount of protein and carbohydrates were also improved, along with increases in dry matter quantification. Two consecutive extraction steps were recommended: One was the separation of water-soluble components such as protein and carbohydrates, and the next step was the extraction of hydrophobic components (pigments). PEF was applied on fresh microalgae *Auxenochlorella protothecoides*, and treated suspensions were incubated under inert conditions. As a result, a larger number of ions and carbohydrates were released and increased the yields of subsequent lipid extraction. 

In addition, total liquid extractions were achieved by applying only 0.25 MJ kg^−1^ DW energy. Most of the extraction process was based on solar drying. However, weather dependency and process controllability were the main limitations [132]. To overcome these limitations, an alternative process was developed to extract the microalgae products with no need for the drying process. Application of a constant electric field was used to extract the nutrients from the microalgae and control the contaminants in microalgae cultivation. The effect of PEF on the cell wall of the microalgae was investigated [133]. The degree of mechanical stability was compared with untreated biomass under high-pressure homogenization. PEF was applied as a pre-treatment step on the unwashed microalgae suspension [134]. The conductivity was reduced by a factor of eight and extraction efficiency was increased by 47%. Finally, this work reveled that at lower conductivity in PEF treatment, a higher efficiency could be achieved in terms of energy. A new door has been opened for hydrocarbon extraction from microalgae in terms of money and energy [135]. Medium conductivity and maturation of algae highly affected the extraction efficiency [136]. Here, *B. braunii Kutzing* showed greater susceptibility than *B. braunii* in PEF cell hatching. 

The effectiveness of nanosecond PEF was revealed as a physical method to extract hydrocarbon from *B. braunii* considering variation of the electric field, duration of the pulse, frequency, and number of pulses. The experimental results represented that an energy consumption of 16.7 J mL^−1^ with 50 pulses were the most suitable parameters for oil extraction [126]. The factors affecting the PEF-assisted extraction—biomass concentration, number of PEF pulses, and initial temperature of algae suspension—were evaluated for the production of crude aqueous extract from *Laminaria digitata* [137]. The supernatant yield was 70 ± 15% and 15 ± 8% for a temperature of −1 to 13 °C, respectively. The higher the yields, the lower the concentration of biomass, and the number of pulses was positively correlated with the temperature change during the PEF treatment and vice versa at the initial temperature. The extraction of pigments, proteins, carbohydrates, and ionic components from *P. kessleri* was examined by the simultaneous application of PEF treatment and HPH treatment [138]. The PEF treatment enhanced the extraction of proteins and carbohydrates while reducing total energy consumption, whereas the HPH treatment was suitable for simultaneous release of all the bioproducts. 

The potential of PEF has been investigate for the extraction of proteins, oils, and carbohydrates from *Arthrospira maxima* [139].This study showed that more pure yields could be produced by PEF treatment, and *C-phycocyanin* extractions were increased by 90% in comparison to bead milling. The effects of PEF have been investigated with hydrothermal liquefaction considering no PEF-treated algae, PEF-treated algae, no PEF-treated lipid extracted algae, and PEF-treated lipid extracted algae [140]. A notable development was obtained in terms of lipid extraction yield, protein extraction, and biocrude. A PEF-aided cascade method was proposed for the lipid extraction of *Scenedesmus almeriensis* [141]. Firstly, carbohydrates were collected in water fraction, followed by protein enzymatic hydrolysis and finally the lipid extraction. A total of 70% lipids were extracted by cascade treatment, and the incubation process increased this percentage to 83%.

## 3. Pulsed Electric Field-Based Extractions 

**Figure 4:**
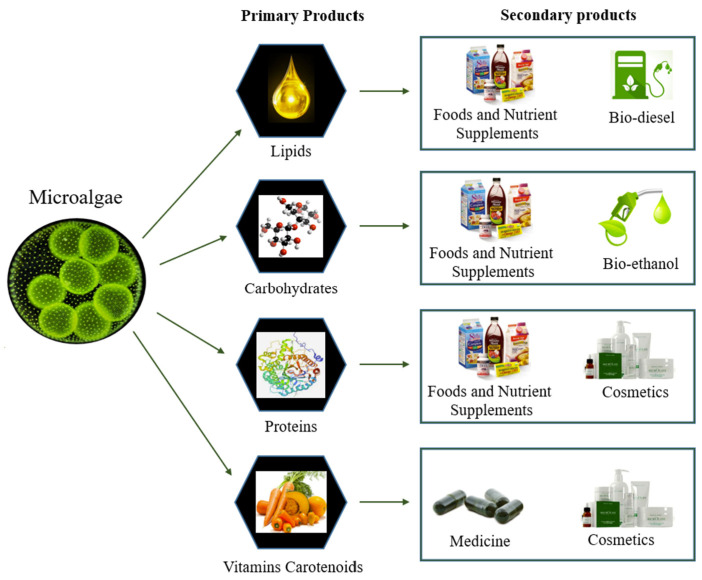
Primary components that can be extracted from microalgae and their secondary products.

As discussed earlier, a variety of factors influence the amount of microalgal products, including the species of microalgae, cultivation conditions, cell disintegration methods, and extraction techniques. After reviewing the current methods for disrupting microalgal cells, we focus our attention on the products that can be obtained from microalgae by means of an effective extraction technique. For microalgae extraction, pulsed electric fields have been found to be an increasingly attractive technology owing to their effectiveness as well as their simplicity [142]. Therefore, PEF-assisted microalgal compound extraction needs to be discussed further in order to fully understand its potential within this market. As a source of proteins [143], hydrocarbons [144], and other nutrients [145], microalgae can also be utilized to produce secondary products from carbohydrates such as bioethanol [146], biogas, biohydrogen [147], and biodiesel [148]. The components that can be extracted from microalgae and their secondary products are shown in Figure 4. 

### 3.1. Protein Extraction

Microalgae and seaweed are deliberated as a practicable source of protein. Some species of microalgae and seaweed contain comparable amounts of protein to other well-known sources, such as milk, eggs, meat, and soybeans [149]. In terms of nutrition value and productivity, protein from algae has numerous advantages over the traditional high-protein crops. Microalgae has more protein yield (4–5 tons/Ha/year) per unit area, whereas traditional crops like wheat, soybean, and pulse legumes produce 1.1 tons/Ha/year, 0.6–1.2 tons/Ha/year, and 1–2 tons/Ha/year, respectively [150]. Pulsed electric fields were applied for protein extraction from *C. reinhardtiithe* microalgae [151]. In that study, the authors found that the outer cell wall of microalgae was not completely fragmented, but rather opened by PEF. In addition, pretreatment along with lower energy input enhanced the protein release. Furthermore, enzymatic-based PEF (E-PEF) has also been recommended as an upright and favorable technology for microalgal cell disruption. 

During the initial period of PEF application, two different types of microalgae, *Chlorella vulgaris* and *Neochloris oleoabundans*, were considered for protein extraction from microalgae [152]. However, only 13% protein was obtained from this research, and the amount of protein remained unchanged even after the input energy was increased. Similarly, for *Nannochloropsis gaditana*, the extraction efficiency followed a declining route along with the high energy cost per unit of protein released in the PEF-based treatment [153]. 

Low PEF was applied for 10 min for the extraction of protein from *Chlorella vulgaris*, *Chlorococcum* sp., and *Scenedesmus* sp. [154]. This study recommended a field strength of 80 V cm^−1^, a duty cycle of 32%, and a temperature of 25 °C as the best extraction parameters. The longer application of PEF increased the inhomogeneity of the algal samples and further created a non-uniform field. Finally, this non-uniform field led to more protein extraction. In PEF-assisted treatment, the extraction efficiency depends on the cell growth, and protein extraction increases with increasing field strength [155]. Moreover, the extraction of protein using PEF is probably limited by diffusion. The effects of the processing parameters of PEF were investigated for the extraction of microalgal compounds from *A. platensis* [156]. The application of a monopolar pulse during the PEF reflected better protein extraction than a bipolar pulse, and it depended on the delay time. Furthermore, the application of mild heating along with the PEF treatment is recommended for the efficient recovery of microalgal compounds. The extraction yields were independent of the strength of the field and pulse frequency [157]. The performance of PEF on the extraction of protein from *A. platensis* was compared with the combined application of PEF and high shear homogenization [158]. The cascade treatment consumed less energy and produced more extracting compounds. A continuous microsecond pulsed electric field (µsPEF) was introduced for the extraction of protein aggregation from microalgae [159]. The mechanism of µsPEF-triggered extractions was related to the production from thermal treatment. It was concluded that thermal effects were mostly responsible for aggregation shifts in the final products. In Table 5, the effects of various parameters in PEF-assisted treatment on protein extraction from different species of microalgae are summarized.

### 3.2. Extraction of Carbohydrates and Their Products

Nowadays, microalgae are measured as the utmost hopeful renewable feedstock for the production of biofuel and biorefinery, owing to their benefits of a fast growing rate, and the fascination with efficient carbon dioxide without depleting the arable land and drinkable water [166]. In addition, for the treatment of wastewaters, algae can be used in agro-industries. Algae can be cultivated in wastewaters, and the capture of nutrients are done along with the conversion of organic matter into algal biomass [167]. These biomasses can be used for the production of biofuels and biochemicals such as biodiesel [168] and bioethanol [169]. Eventually, environmental and economic advantages can be achieved. Since microalgal-based carbohydrates are mostly in the form of starch and cellulose, it is easy to convert them into monosaccharides compared to lignocellulosic resources [170]. Microalgae such as *Chlorella*, *Spirulina*, *Chlamydomonas*, *Tetraselmis*, and *Scenedesmus* are acknowledged to contain a large amount of carbohydrates (>50% of the dry weight) [169]. At present, PEF treatment is considering a promising alternative to some conventional carbohydrate extraction techniques, i.e., bead milling and homogenization. PEF treatment is applied for the extraction of intracellular components like carbohydrates from *Chlorella vulgaris* considering the various process parameters [131]. Gianpiero Pataro et al. [171] suggested that increasing the input energy in the PEF treatment is better than increasing the field strength for stimulating the leakage of intraocular compounds. The PEF treatment is proposed as a prospective technology for the extraction of carbohydrates and protein from *C. vulgaris* TISTR8580. Increasing the PEF pulses increases the conductivity and temperature of the algal extract, and the highest amount of carbohydrates (23.19 ± 1.47 mg L^−1^) can be obtained by applying 2500 pulses.

Nanosecond pulsed electric field (nsPEF) treatment is introduced as an innovative, resource-efficient, and technology-driven technique for transferring microalgae intracellular compounds into possible raw materials for bio-based industries [172]. Three treatment parameters (10 kV cm^−1^, pulse width: 100 ns, and frequency: 5 Hz) were set for nsPEF treatment and 17.53 ± 10.46% of biomass was extracted. However, protein extraction using this technique is discouraged. The extraction of carbohydrates using PEF and ultrasonication was examined and compared for three types of microalgae (*Nannochloropsis* sp., *Phaeodactylum tricornutum*, and *Parachlorallea kessleri*) [173]. This work showed that the performance of the extraction for both the treatments depends on the applied treatment methods and microalgal species. To obtain the maximum amount of biomolecules in PEF treatment, the intensive disruption technique is recommended. A carbohydrate-rich microalgae species (*Saccharomyces cerevisiae*) was studied for the production of bioethanol using PEF-assisted pre-treatment and a fermentation process [146]. Here, 53.08% of cellulose content was obtained after treatment and it proved the suitability for the production of bioethanol. A novel nanosecond pulsed electric field (nsPEF)-assisted technique was offered for continuous hydrocarbon extraction with a cost-effective approach [174]. Oil was extracted by optimizing the values of electric field, energy spent, and pulse repetition frequencies (65 kV cm^−1^, 55.6 J mL^−1^, and 500 Hz, respectively) from microalgae matrix rather than the cells. Hydrocarbon extraction efficiency depends on the selection of the medium of extraction considering the pulse duration and applied voltage [136]. AF_6_ medium is better than CHU_13_ medium in the case of the efficiency of extraction. In Table 6, the effects of the parameter variation of the PEF-assisted treatment on carbohydrate extraction from various species of microalgae are summarized.

### 3.3. Lipid Extraction

Global population growth has resulted in an increase in the demand for eco-friendly feedstock, alternative fuels, and pharmaceuticals [175]. As a result, the exploration of renewable resources has grown increasingly important. Microalgae have been recognized as an incredible source of lipids and carotenoids. Among lipid molecules are vitamins (such as A, D, E, and K), fats, waxes, hormones, and oils. Microalgal lipids can be divided into two categories based on the number of carbon atoms present. The first is fatty acids, which contain 14–20 carbons and are used to produce biodiesel. Polyunsaturated fatty acids (PUFAs) are another type of fatty acid with more than 20 carbon atoms, and are used for food and nutritional supplements. These include docosahexaenoic acid and eicosapentaenoic acid [176]. In microalgae, the amount and composition of lipids are primarily determined by species, and can be influenced by external factors such as temperature, metal stress, nutrient starvation, light intensity, and salinity stress [177]. For the extraction of lipids from microalgae, PEF-assisted treatment has become an alternative pretreatment technique since this pretreatment permits up to 97% of the lipid extraction yield to be obtained [178]. PEF-assisted extraction was applied for the extraction of intracellular lipids from microalgae *Synechocystis* PCC 6803 [179]. Keeping the same extraction efficiency, PEF required lower usage of isopropanol and increased the prospect for the low-toxicity solvent isopropanol to obtain lipid particles during the extraction. A low-energy pulsed electric field (LE-PEF) was introduced to improve the lipid productivity of *Acutodesmus dimorphus* microalgae [180]. The LE-PEF on–off cycle treatment was optimized for *A. dimorphus* (2 s on, 60 s off for 15 min, 6 cycles/day) and an overall 28.8% lipids were extracted. Lipids were extracted using PEF pretreatment from *Chlorella pyrenoidosa* microalgae in wastewater [181]. After the pretreatment, the extracted yields as fatty acid methyl esters were compared with the ultrasonication assisted technique, and the PEF-assisted method dominated with a 12% higher lipid production. 

PEF-assisted lipid extraction can be performed on wet biomass. Hence, it does not need an energy-consuming drying step. In optimized conditions, the input energy requirement for PEF pretreatment is 1.5 MJ kg^−1^ of dry matter [178], whereas this requirement is less for bead milling, at 1.8 MJ kg^−1^ DW [57]. However, this is not the lowest energy requirement, because by using the HPH technique, only 0.16 MJ kg^−1^ DW input energy is required for extraction from *Tetraselmis suecica* microalgae [182]. An energy-efficient approach considering the combination of the PEF-assisted technique and hydrothermal liquefaction (HTL) was proposed for the extraction of hydrolysis–amino acids, lipids, and protein from different microalgae species such as *Auxenochlorella protothecoides*, *Chlorella vulgaris*, and *Scenedesmus almeriensis* [183]. The PEF treatment was able to improve the lipid extraction yield by 29% and reduce the biocrude yield by 15%. Moreover, energy consumption was reduced to 0.25 MJ kg^−1^ DW. The field strength of the PEF-assisted process was changed to investigate the extraction performance of lipids in fresh *C. sorokiniana* suspension [165]. Unfortunately, the PEF treatment was unable to contribute to lipid extraction for *C. sorokiniana*. On the other hand, the lipid extraction yield from *Chlorella* mainly depended on the electric field strength [184]. An effective approach was developed for lipid extraction from wet *Scenedesmus almeriensis*, where 1.5 MJ kg^−1^ DW input energy was required for PEF treatment. Lipids were extracted with a combination of ethanol and hexane as co-solvent. Almost 70% of the total lipids could be extracted using the PEF-assisted treatment, whereas 43% of lipids were extracted from the untreated biomass [141]. Table 7 demonstrates the effects of the parameter variation of the PEF-assisted treatment on the extraction of lipids from various microalgae species.

## 4. Discussion

Over the course of decades, many research projects have been conducted to enhance the performance of existing methods. These studies have also helped to gain a deeper understanding of the challenges faced by this emerging field. An overview of these studies provided in this review article summarizes the ongoing challenges and progress made in this area. Due to the wide range of research topics available within this area, however, this review is primarily focused on methods of obtaining microalgal intracellular compounds. For this purpose, we provided an overview of the currently available methods of cell disruption as the first step of the extraction process. Here, the most important technical parameters related to the advantages of these methods were outlined. Following the collection of data from various studies, we then compared the efficacy of the different cell disruption methods based on the type of microalgae used in their studies. These comparative data are presented in multiple tables throughout the article. Analysis of the information provided in the tables shows how the studies have attempted to optimize their methods by adjusting a number of variables, which may have had an impact on the end results of the harvesting process. As a final step, we evaluated the limitations of each method in light of potential future applications. 

As a result, it was shown that the production of industrial-scale biofuels from microalgae requires an efficient recovery and purification scheme for carbohydrates and lipids [187]. In addition, the efficiency of another hopeful technique for lipid extraction, the enzymatic disruptive technique, is limited to the type of the microalgae and the lipid composition [44]. Furthermore, the cost of enzymic extraction is high, which limits the usage of enzyme hydrolysis on an industrial scale [188]. Mechanical cell disruption techniques, including bead milling, high-pressure homogenization, steam explosion, and ultrasonication, are encouraging technologies for commercial-scale implementation, offering higher efficiency and providing extracts with a high level of purity. Bead beating was shown to be simple and fast, with a high reproducibility rate and low labor cost [94]. However, scale-up is difficult in the bead-milling-assisted pretreatment of microalgal cells, and a cooling jacket is required to avoid the degradation of the required product [189]. Another alternative mechanical technique, such as ultrasonication, on the other hand, can be operated at low temperature, reduces extraction time, and makes it easier to release the intracellular components [190]. However, the ultrasound-assisted method requires higher input power, and its species sensitivity and practicability on an industrial scale is not clear [191].

Among different mechanical techniques, the pulsed electric field (PEF) technique has shown significant energy efficiency in cell disruption, with 50%, 80%, and 95% energy saving over mechanical, enzymatic, and thermal pre-treatment techniques, respectively [192]. Moreover, this method does not need to dewater or a drying route process, and thus operational cost is reduced. Furthermore, it is highly scalable, appropriate to treat dry or wet microalgae, and has a lower treating time, and additional chemicals are not mandatory in the PEF-assisted cell disruption method [193]. In addition, PEF treatment can be used in both upstream and downstream techniques for enhancing the extraction yields [194]. In general, there are factors that can affect the extraction yields in PEF-assisted techniques, including the strength of applied electric field, pulse duration, operational time, frequency, physicochemical parameters (conductivity, temperature, and pH), and treatment energy [195].

As a result of its effectiveness and ease of use, PEF has gained popularity as a technology for both cell disruption and microalgae extraction. Therefore, as part of this review, we further expanded our discussion on pulse electric field-assisted microalgal extraction. This was done with regard to the type of compounds targeted by PEF. The effect of various parameters, which can enhance the effectiveness of the technique for that particular component, were further discussed in order to gain a better understanding of its potential applications. 

## 5. Conclusions

The field of research on microalgae intracellular compounds has experienced rapid growth in recent years, primarily due to the growing demand for sustainable food and energy sources. Modern advances in the technologies used for the recovery of microalgal compounds have made it possible to harvest intracellular materials such as protein, carbohydrates, and oil with increased efficiency on an industrial scale. The use of these methods, alone or in combination, has also led to the development of various secondary products from the extracted compounds, such as biofuel, medicine, and cosmetics. Despite this, these methods face the challenge of increasing their efficiency in order to harvest the maximum amount of materials from microalgae at low energy costs and with high quality. Added to these challenges are a number of factors that have a substantial effect on the degree of product yield for mass production. These variables include the type of microalgae, cultivation conditions, and the feasibility of cell disintegration and extraction methods on a large scale. 

In spite of the wealth of knowledge and advances accumulated over the years in the field of microalgae compounds, multidisciplinary studies are still necessary to establish a sustainable and scalable system for fully exploring the potential of microalgae as a rich source of material and energy. It is evident that there is a significant information gap to address challenges in various areas of this growing industry, from microalgal cultivation to final product extraction. The first step toward achieving this goal is understanding in detail the challenges and advantages of currently available methods of cell disruption and extraction from a wide range of scientific literature.

## Figures and Tables

**Table 1:** Effects of the parameters of the bead-beating technique for the cell disruption of various types of microalgae. DCW: dry cell weight; DW: dry weight; BD: bead diameter; AS: assisted solvent; FAME: fatty acid methyl esters.

Microalgae	Parameters of Bead-Beating Technique	Extracted Products (%)	Reference
*Nannochloropsis gaditana*	BD: 0.4 mm, DW: 10 g kg^−1^, rotation: 10 m s^−1^	Lipid (17.7%)	[59]
*Chlorella vulgaris*	BD: 0.4 mm, DW: 25 g L^−1^, 10 min, 2039 rpm	Lipid (35%)	[45]
*Tetraselmis suecica*	BD: 0.4 mm, DW: 100 g L^−1^, 30 min, 2039 rpm	Lipid (17.6%)	[60]
*1012Scenedesmus* sp. (*Chlorophyceae*)	BD: 0.1 mm, DCW: 0.5 g L^−1^, 5 min, 2800 rpm	Lipid (9%)	[61]
*Botryococcus* sp. (*Trebouxiophyceae*)	BD: 0.1 mm, DCW: 0.5 g L^−1^, 5 min, 2800 rpm	Lipid (28%)	[61]
*Chlorella vulgaris* (*Trebouxiophyceae*)	BD: 0.1 mm, DCW: 0.5 g L^−1^, 5 min, 2800 rpm	Lipid (8%)	[61]
*Tetradesmus dimorphus* (*Chlorophyceae*)	DCW: 40 g L^−1^, 5 min, borosilicate glass balls: 0.1 mm	FAME (20%)	[62]
*Chlorella* sp. (*Trebouxiophyceae*)	Chamber: 240 mL, 160 mL, 20 min, 1500 rpm, glass beads: 0.40–0.60 mm	Lipid (10%)	[63]
*Phaeodactylum tricornutum* (*Bacillariophyceae*)	DCW: 100 mg, 10 min, 2000 rpm	Lipid (39%)	[64]
*C. sorokiniana* (*Trebouxiophyceae*)	DCW: 25 g L^−1^, 5 min, 2800 rpm	Lipid (6.6%)	[65]
*Chlorella. vulgaris* (*Trebouxiophyceae*)	BD: 1–1.6 mm, 1–30 min, 2500 rpm	Protein (2–50%)	[66]
*Chlorella. vulgaris* (*Trebouxiophyceae*)	DCW: 25–145 g kg^−1^, AS: 6–12 m s^−1^, 3 min	Protein (32–42%)	[67]
*C. vulgaris* (*Trebouxiophyceae*)	DW: 40 mg, speed: 1/30 s, 25 min	Lipid (40%)	[68]
*C. vulgaris* (*Trebouxiophyceae*)	DW: 7.7%, 1 h, 2500 rpm	Lipid (10%)	[69]

**Table 2:** Effects of the parameters of the microwave irradiation technique for the cell disruption of various types of microalgae. DCW: dry cell weight; DW: dry weight; WB: wet biomass; F: frequency; T: temperature; B/S: biomass/solvent; FAME: fatty acid methyl esters.

Microalgae	Parameters of Microwave Technique	Extracted Products (%)	Reference
*Nannochloropsis gaditana*	DW: 10 g kg^−1^, T: 91 °C, 5 min	Lipid (49%)	[59]
*Scenedesmus* sp. (*Chlorophyceae*)	DCW: 0.5 g mL^−1^, T: 100 °C, 5 min, F: 2450 MHz	Lipid (11%)	[61]
*Botryococcus* sp. (*Trebouxiophyceae*)	DCW: 0.5 g mL^−1^, T: 100 °C, 5 min, F: 2450 MHz	Lipid (29%)	[61]
*Chlorella vulgaris* (*Trebouxiophyceae*)	DCW: 0.5 g mL^−1^, T: 100 °C, 5 min, F: 2450 MHz	Lipid (10%)	[61]
*Scenedesmus* sp. (*Chlorophyceae*)	DCW: 50 g L^−1^, T: 100 °C, 10 min, 100 W	Lipid (28.33%)	[87]
*Tetradesmus dimorphus* (*Chlorophyceae*)	DCW: 40 g L^−1^, T: 100 °C, 5 min	FAME (80%)	[88]
*Chlorella* sp. (*Trebouxiophyceae*)	WB: 5 g L^−1^, T: 100 °C, 5 min, F: 2450 MHz	Lipid (18%)	[63]
*Nannochloropsis gaditana* (*Eustigmatophyceae*)	T: 60–90 °C, 10–20 min, 25–35 W	Lipid (30–40%)	[89]
*Nannochloropsis* (*Eustigmatophyceae*)	DW: 1 g, 5 min cycle mode, on: 21 s, off: 9 s, F: 2450 MHz	FAME (37%)	[90]
*Nannochloropsis oculata* (*Eustigmatophyceae*)	DCW: 2 g, T: 100 °C, 5–15 min, F: 2455 MHz, 900 W	Lipid (17–27%)	[91]
*Nannochloropsis gaditana* (*Eustigmatophyceae*)	WB: 20 mL, T: 150 °C, 5 min, F: 2450 MHz	Lipid (11.2%)	[92]
*C. sorokiniana* (*Trebouxiophyceae*)	WB: 20 mL, T: 150 °C, 5 min, F: 2450 MHz	Lipid (14.5%)	[92]
*Phaeodactylum tricornutum* (*Bacillariophyceae*)	WB: 20 mL, T: 150 °C, 5 min, F: 2450 MHz	Lipid (27.1%)	[92]
*Chlorococcum* sp. (*Chlorophyceae*)	DCW: 20 g L^−1^, T: 100 °C, 2–6 min, F: 2450 MHz	Lipid (18–24%)	[30]
*Botryococcus* sp. (*Trebouxiophyceae*)	DCW: 20 g L^−1^, T: 100 °C, 2–6 min, F: 2450 MHz	Lipid (22–48%)	[30]
*C. sorokiniana* (*Trebouxiophyceae*)	DCW: 20 g L^−1^, T: 100 °C, 2–6 min, F: 2450 MHz	Lipid (11–35%)	[30]
*C. sorokiniana* (*Trebouxiophyceae*)	DCW: 25 g L^−1^, T: 100 °C, 5 min, F: 2450 MHz	Lipid (0.9%)	[65]
*C. vulgaris* (*Trebouxiophyceae*)	B/S: 1/100, T: 40–50 °C, 5–30 min, 300 W	Lipid (70–72%)	[93]

**Table 3:** Effects of the parameters of the ultrasonication technique for the cell disruption of various types of microalgae. DW: dry weight; DCW: dry cell weight; WB: wet biomass; T: temperature; B/S: biomass/solvent; TAG: triacylglycerol; FAME: fatty acid methyl esters; F: frequency).

Microalgae	Parameters of Sonication Technique	Extracted Products (%)	Reference
*Nannochloropsis gaditana*	DW: 10 g kg^−1^, F: 20 kHz, 30 min, 130 W	Lipid (21.7%)	[59]
*Scenedesmus* sp. (*Chlorophyceae*)	DCW 0.5 g L^−1^, 5 min, F: 10 kHz	Lipid (6%)	[61]
*Botryococcus* sp. (*Trebouxiophyceae*)	DCW: 0.5 g L^−1^, 5 min, F: 10 kHz	Lipid (9%)	[86]
*Chlorella vulgaris* (*Trebouxiophyceae*)	DCW: 0.5 g L^−1^, 5 min, F: 10 kHz	Lipid (6%)	[86]
*Scenedesmus* sp. (*Chlorophyceae*)	DCW: 50 g L^−1^, 2 min, F: 15 kHz	Lipid (19%)	[87]
*Scenedesmus* sp. (*Chlorophyceae*)	DCW: 2 g L^−1^, 30 min, 100 W	Lipid (6%)	[111]
*Spirulina platensis*	DCW: 0.4 g L^−1^, 5 min, F: 24 kHz, 400 W	Proteins (27%)	[102]
*Nannochloropsis gaditana*	DCW: 0.4 g L^−1^, 5 min, F: 24 kHz, 400 W	Lipids (41%), Carbohydrates (33%)	[102]
*Scenedesmus almeriensis*	DCW: 0.4 g L^−1^, 5 min, F: 24 kHz, 400 W	Carbohydrates (44%), proteins (32%)	[102]
*Tetradesmus dimorphus* (*Chlorophyceae*)	DCW: 40 g L^−1^, 30 min, F: 50/60 Hz	FAME (73%)	[88]
*Nannochloropsis oceanica*	WB 1 mL, 120 min F: 20 kHz	Lipid (98.2%)	[112]
*Neochloris oleoabundans* (*Chlorophyceae*)	DCW: 20 g L^−1^, 30 min, F: 25 kHz, 600 W	Lipid (4 g L^−1^)	[113]
*Nannochloropsis oculata* (*Eustigmatophyceae*)	DW: 100 g, 360 min, 30 kHz	Lipid (24.3 g)	[114]
*Nannochloropsis gaditana* (*Eustigmatophyceae*)	5–20 min, T: 50–60 °C, F: 19.5 kHz, 100 W	Lipid (31–38%)	[89]
*Nannochloropsis* (*Eustigmatophyceae*)	DCW: 1 g, T: 50–60 °C, 5 min, F: 20 kHz	FAME (21%)	[90]
*Nannochloropsis* sp. (*Eustigmatophyceae*)	DW: 1 g, 5 min, interval: 1 min, F: 50 Hz, 30 W	Lipid (22.5%)	[115]
*Nannochloropsis oculata* (*Eustigmatophyceae*)	DCW: 3 g, 5–15 min, T: 50 °C, F: 24 kHz	Lipid (28–30%)	[91]
*Nannochloropsis gaditana* (*Eustigmatophyceae*)	WB: 20 mL, 5 min, F: 37 kHz	Lipid (10.5%)	[92]
*C. sorokiniana* (*Trebouxiophyceae*)	WB: 20 mL, 5 min, F: 37 kHz	Lipid (14.1%)	[92]
*Phaeodactylum tricornutum* (*Bacillariophyceae*)	WB: 20 mL, 5 min, F: 37 kHz	Lipid (28%)	[92]
*Phaeodactylum tricornutum* (*Bacillariophyceae*)	DCW 100 mg, 15 min, T: 40 °C	Lipid (38%)	[64]
*C. sorokiniana* (*Trebouxiophyceae*)	DCW 25 g L^−1^, 10 min, F: 10 kHz	Lipid (4.4%)	[65]
*C. vulgaris* (*Trebouxiophyceae*)	5–30 min, on at 5 s, off for 15 s, F: 20 kHz	Protein (4–9%)	[66]
*C. vulgaris* (*Trebouxiophyceae*)	B/S:1/100, 5–30 min, 300 W, T: 40–50 °C, F: 40 kHz	Lipid (70–80%)	[93]
*C. vulgaris* (*Trebouxiophyceae*)	DCW: 2.68 g L^−1^, 20 min, interval: 5 s and 30 s, 600 W	Lipid (15%)	[63]
*C. vulgaris* (*Trebouxiophyceae*)	DCW: 18%, pH: 5.8, T: 37 °C, concentration: 0.5–8%	Lipid (19–35%)	[44]
*C. vulgaris* (*Trebouxiophyceae*)	DW: 40 mg, 25 min, 400 W	Lipid (35%)	[68]
*Chaetoceros ceratosporus* (*Mediophyceae*)	30 min, T: 5 °C, 50 W	TAG: 1.5 to 3 μg	[116]

**Table 4:** Effects of the parameters of the autoclaving technique for the cell disruption of various types of microalgae. DW: dry weight; DCW: dry cell weight; WB: wet biomass; T: temperature.

Microalgae	Parameters of Autoclaving Technique	Extracted Products (%)	Reference
*Scenedesmus* sp.	DCW: 0.5 g L^−1^, 1.5 MPa, 5 min, T: 125 °C	Lipid (5%)	[61]
*Botryococcus* sp.	DCW: 0.5 g L^−1^, 1.5 MPa, 5 min, T: 125 °C	Lipid (12%)	[61]
*Chlorella vulgaris*	DCW: 0.5 g L^−1^, 1.5 MPa, 5 min, T: 125 °C	Lipid (10%)	[61]
*Chlorella* sp. (*Trebouxiophyceae*)	WB: 5 g L^−1^, Pressure: 15 lbs. in^−2^, 5 min, T: 121 °C	Lipid (0.11 g)	[63]
*Chlorella* sp. (*Trebouxiophyceae*)	20 min, T: 105 °C	Lipid (27%)	[122]
*Nannochloropsis oculate*	DW: 2 g, Pressure: 15 lbs. in^−2^, 10–30 min, T: 121 °C	Lipid (28–29%)	[91]
*Nannochloropsis gaditana*	WB: 20 mL, Pressure: 29 lbs. in^−2^, 5 min, T: 120 °C	Lipid (10.8%)	[92]
*C. sorokoniana*	WB: 20 mL, Pressure: 29 lbs. in^−2^, 5 min, T: 120 °C	Lipid (14.4%)	[92]
*Phaeodactylum tricornutum*	WB: 20 mL, Pressure: 29 lbs. in^−2^, 5 min, T: 120 °C	Lipid (27.2%)	[92]
*Chlorococcum* sp.	DCW: 20 g L^−1^, 1.5 MPa, 15 min, T: 121 °C	Lipid (15–25%)	[30]
*Botryococcus* sp. (*Trebouxiophyceae*)	DCW: 20 g L^−1^, 1.5 MPa, 15 min, T: 121 °C	Lipid (38–48%)	[30]
*C. sorokiniana* (*Trebouxiophyceae*)	DCW: 20 g L^−1^, 1.5 MPa, 15 min, T: 121 °C	Lipid (15–32%)	[30]
*C. sorokiniana* (*Trebouxiophyceae*)	DCW: 25 g L^−1^, 15 min, T: 121 °C	Lipid (0.6%)	[65]

**Table 5:** Effect of parameter variation of PEF on protein extraction. DW: dry weight, DCW: dry cell weight; T: temperature; FS: field strength; IE: input energy).

Microalgae	PEF Parameters	Extracted Protein (%)	Reference
*A. platensis*	FS: 10–30 kV cm^−1^, IE: 20–100 kJ kg^−^^1^ sus, pulse delay: 1–20 µs, T: 25 °C	17.4%	[156]
*Chlorella vulgaris* and *Neochloris oleoabundans*	FS: 7.5–130 kV cm^−1^, Pulses: 1–40, pulse delay: 0.05–5 ms pulses: 1–40, pulse delay: 0.05–5 ms, IE: 0.05–150 kWh kg^−^^1^ DW	13%	[152]
*Nannochloropsis gaditana*	FS: 30 kV cm^−1^, DCW: 15–60 g L^−1^, Pulses: 2–10, pulse interval: 5 s, Pulse delay: 5 ms	10%	[153]
*Nannochloropsis salina*	FS: 3–6 kV cm^−1^, IE: 15.4–30.9 kWh kg^−1^, T: 37 °C	12%	[160]
*Auxenochlorella prothecoides*	FS: 23–43 kV cm^−1^, Pulse delay: 5 ns, IE: 0.15–0.6 kWh kg^−1^, T:14–22 °C	DCW: 2 µg L^−1^	[124]
*Nannochloropsis salina*	FS: 20 kV cm^−1^, IE: 13.3–53.1 kJ kg^−1^, pulse delay: 1–4 ms	3.6%	[161]
*Nannochloropsis salina*	IE: 0.02–14 kWh kg^−1^, T: 5.74–34.45 °C	10%	[162]
*Chlorella vulgaris*	IE: 0.55–1.1 kWh kg^−1^, flow rate: 33 mL min^−1^, T: 25–65 °C	4.9%	[163]
*Chlorella vulgaris*	FS: 10–30 kV cm^−1^, IE: 20–100 kJ kg^−1^ sus, flow rate: 33.3 mL min^−1^, T: 25 °C	5.2%	[164]
*Chlorella vulgaris*	FS: 10–20 kV cm^−1^, IE: 1.94 kJ kg^−1^ sus	29.1%	[155]
*C. sorokiniana*	FS: 5–15 kV cm^−1^, IE: 25–150 kJ kg^−1^, T: 20 °C	5.6%	[165]
*A. platensis*	FS: 20 kV cm^−1^, IE: 100 kJ kg^−1^ sus	8.1%	[158]

**Table 6:** Effect of the parameter variation of PEF on carbohydrate extraction. T: temperature; FS: field strength; IE: input energy.

Microalgae	PEF Parameters	Extracted Carbohydrate (%)	Reference
*A. platensis*	FS: 10–30 kV cm^−1^, IE: 20–100 kJ kg^−1^ sus, pulse delay: 1–20 µs, T: 25 °C	10.1%	[156]
*platensis*	FS: 20 kV cm^−1^, IE: 100 kJ kg^−1^ sus	13.7%	[158]
*Laminaria digitata*	Pulses: 12–268, T: 12–48 °C	15%	[137]
*Chlorella vulgaris*	FS: 27–35 kV cm^−1^, T: 20–25 °C, IE: 50–150 kJ kg^−1^	8%	[131]
*Chlorella vulgaris*	FS: 5 kV cm^−1^, T: 7 °C, Pulses: 1500–4500	23.19 mg L^−1^	[171]
*Chlorella vulgaris*	FS: 10 kV cm^−1^, F: 5 Hz, pulsed delay: 100 ns	17.53%	[172]
*Nannochloropsis* sp.	FS: 20–40 kV cm^−1^, IE: 704 kJ kg^−1^, pulses: 1–800, T: 20 °C	20.2%	[173]
*Phaeodactylum tricornutum*		19.7%	
*Parachlorallea kessleri*		11.1%	

**Table 7:** Effect of the parameter variation of PEF on lipid extraction. DW: dry weight; T: temperature; FS: field strength; IE: input energy.

Microalgae	PEF Parameters	Extracted Lipids (%)	Reference
*Scenedesmus*	IE: 30.6–33.7 kWh m^−3^, T: 13.5–36 °C	21%	[127]
*Acutodesmus dimorphus*	FS: 10 kV cm^−1^, 10 pulses s^−1^, T: 20–25 °C	28.8%	[180]
*Chlorella pyrenoidosa*	FS: 20 kV cm^−1^, F: 150 Hz, pulse width: 0–10 µs	128 mg g^−1^	[181]
*Auxenochlorella protothecoides*	FS: 4 MV m^−1^, pulse delay: 1 µs, IE: 150 kJ kg^−1^, F: 5 Hz,	30–35%	[178]
*A. protothecoides*	IE: 1.5 MJ kg^−1^, T: 15 °C, pulse delay: 1 µs	39.8%	[183]
*Chlorella* sp.	FS: 20–55 kV cm^−1^, pulse delay: 10 µs, flow rate: 50 mL min^−1^, F: 50 Hz	45.7%	[184]
*Scenedesmus almeriensis*	IE: 1.5 MJ kg^−1^ DW, T: 20–33 °C	43%	[141]
*Auxenochlorella protothecoides*	FS: 35 kV cm^−1^, IE: 1.5 MJ kg^−1^ DW, pulse delay: 1 µs	20%	[75]
*Auxenochlorella protothecoides*	FS: 23–43 kV kg^−1^, IE: 52–211 kJ kg^−1^, pulse delay: 20 ns	20%	[124]
*Chlorella vulgaris*	FS: 5 kV cm^−1^, pulse delay: 10 µs–10 ms	22%	[185]
*C. vulgaris*	FS: 20 kV/cm, IE: 100 kJ kg^−1^ SUS, T: 25 °C	21%	[186]

## Data Availability

Data are contained within the article.
